# Analysis of differential expression of matrix metalloproteinases and defensins in the nasopharyngeal milieu of mild and severe COVID-19 cases

**DOI:** 10.1371/journal.pone.0304311

**Published:** 2025-02-18

**Authors:** Khekashan Imtiaz, Nida Farooqui, Khalid Ahmed, Alnara Zhamalbekova, Muhammad Faraz Anwar, Asghar Nasir, Zeeshan Ansar, Khitab Gul, Azhar Hussain, Antonio Sarría-Santamera, Syed Hani Abidi

**Affiliations:** 1 Department of Pathology and Laboratory Medicine, Aga Khan University, Karachi, Pakistan; 2 Department of Biological and Biomedical Sciences, Aga Khan University, Karachi, Pakistan; 3 Nazarbayev University School of Medicine, Astana, Kazakhstan; 4 Department of Biochemistry, Bahria University Medical and Dental College, Karachi, Pakistan; 5 Department of Biosciences, Muhammad Ali Jinnah University, Karachi, Pakistan; 6 Department of Biomedical Sciences, Nazarbayev University School of Medicine, Astana, Kazakhstan; University of Hyderabad, INDIA

## Abstract

**Introduction:**

A subset of COVID-19 disease patients suffers a severe form of the illness; however, underlying early pathophysiological mechanisms associated with the severe form of COVID-19 disease remain to be fully understood. Several studies showed the association of COVID-19 disease severity with the changes in the expression profile of various matrix metalloproteinases (MMPs) and defensins (DA). However, the link between the changes in the expression of MMPs and DA in the nasopharyngeal milieu during early phases of infection and disease severity remains poorly understood. Therefore, we performed differential gene expression analysis of MMPs and DA in the nasopharyngeal swab samples collected from normal (COVID-19 negative), mild, and severe COVID-19 cases and examined the association between MMP and DA expression and disease severity.

**Material and method:**

A total of 118 previously collected nasopharyngeal samples from mild and severe COVID-19 patients (as per the WHO criteria) and 10 healthy individuals (COVID-19 negative, controls) were used in this study. A real-time qPCR assay was used to determine the viral loads and assess the mRNA expression of MMPs and DA. One-way ANOVA was applied to perform multiple comparisons (estimate differences) in MMPs and defensin gene expression in the normal vs mild vs severe groups. In addition, a multivariable logistic regression analysis was carried out with all the variables from the data set using ‘severity’ as the outcome variable.

**Results:**

Our results showed that as compared to controls, DA1, DA3, and DA4 expression was significantly (p < 0.05) upregulated in the mild group, whereas the expression of DA6 was significantly downregulated in both mild and severe groups (p-value < 0.05). Similarly, compared to controls, the expression of MMP1 and MMP7 was significantly downregulated in both mild and severe groups, whereas MMP2 expression was upregulated in the mild group (p-value < 0.05). Additionally, the regression analysis showed that the expression of MMP1, MMP2, and MMP9 was significantly associated with the severity of the disease.

**Conclusion:**

The early detection of changes in the expression of MMPs and defensins may act as a useful biomarker/predictor for possible severe COVID-19 disease, which may be useful in the clinical management of patients to reduce COVID-19-associated morbidity and mortality.

## Introduction

SARS-CoV-2, although significantly controlled by vaccine, remains a continuous threat to human health with substantial economic, social, and health implications [1–4]. Previous studies have shown that the SARS-CoV-2 virus has two possible entry routes: via endolysosomal cathepsins and the transmembrane serine protease 2 (TMPRSS2) [5].

The underlying pathophysiological mechanisms that are associated with the severe form of COVID-19 disease and death due to COVID-19-associated complications remain to be fully understood. The pre-existing comorbidities, e.g., diabetes, hypertension, compromised immune system, etc., have been associated with increased severity of COVID-19 [6,7]. For example, when the immune system becomes compromised, it can lead to increased levels of metalloproteinases, specifically gelatinases like MMP2, in the plasma, that may contribute to various pathological processes [8]. Therefore, it has been hypothesized that depending on the age and genetic polymorphisms, the pre-infection level of plasma matrix metalloproteinase (MMPs) or the potential of the host cells to secrete these proteases may be associated with the severe form of COVID-19 disease [9]. MMPs, being proteolytic enzymes, can damage various extracellular matrix components and are associated with the modulation of various cytokines and growth factors [10]. Recent data has also shown that increased plasma MMPs are associated with the severity of COVID-19 disease [11]. Furthermore, COVID-19-associated lung damage has also been shown to be associated with MMPs [12,13], for instance, upregulation of gene expression of MMP2 and MMP9 in COVID-19 patients is associated with increased risk of respiratory failure [14,15]. Additionally, it has been reported that MMPs can facilitate the viral entry and formation of syncytia in the context of dysregulated immune response and hyperinflammation in COVID-19 patients. Therefore, MMPs such as cathepsins and serine proteases can be potential therapeutic targets to treat severe COVID-19 patients.

Similar to MMPs, defensins (antimicrobial peptides) have been proposed as immunologic factors in the pathophysiology of COVID-19 disease and its severity [16,17]. Defensins can expressed by mucosal epithelial cells as part of the innate immune response against the colonization of various pathogens [18]. Mild forms of COVID-19 disease may be associated with effective expression of defensins, as it has been shown that the activity of α- and β-defensins play a major role in controlling upper respiratory tract viral infections [19]. Recently, defensins have been explored as potential antiviral therapeutic agents against SARS-CoV-2; however, the exact relationship between various defensins and SARS-CoV-2 infection, especially during the early days of infection, has yet to be elucidated [20,21].

Thus, a detailed analysis of the expression profile of human defensin genes may be useful to understanding the role of the viral infection patterns, innate immune response, and its subsequent association with the COVID-19 disease. Therefore, we performed differential gene expression analysis of matrix metalloproteinases (MMPs) and defensins in the nasopharyngeal swab samples collected from normal, mild, and severe COVID-19 cases and examined the association between MMP and DA expression and disease severity.

## Methodology

### Sample collection and characterization of the sample as mild and severe based on the patient’s symptoms

This was a retrospective, cross-sectional study performed on a total of 118 SAR-CoV-2 PCR-positive nasopharyngeal swab samples collected between Jan–Dec 2021. The samples from mild and severe patients were collected within three days of infection. The period for the current study, during which the samples were accessed, was Jan 1, 2023, to Sep 30, 2023. Additionally, nasopharyngeal swab samples from ten normal individuals with confirmed SARS-CoV-2 negative PCR were all included in the study. As described previously, using the WHO diagnostic criteria, these samples were characterized as severe and mild based on symptoms observed in the patients [22,23]. The samples were originally collected after obtaining written informed consent from each participant: the samples for the mild group were taken within three days of symptoms appealing, while samples for the severe group were taken within three days of admission to the hospital and stored at -80˚C until further use [22,23]. The study was approved by the Ethics Review Committee, Aga Khan University Hospital (ERC#2021-5456-15382). To ensure the patients’ confidentiality, the samples were given unique IDs. Additionally, the data was collected anonymously and cannot be used to identify the patients.

### RNA extraction, cDNA synthesis, estimation of viral loads, and gene expression analysis of MMP and defensin genes

Viral and total RNA was extracted from all the SARS-CoV-2 positive patient and control (normal) nasopharyngeal samples using a QIAamp RNA kit (Qiagen, Hilden, Germany). For reverse transcription, 2.5ug RNA/20ul of cDNA synthesis reaction was carried out using ONE SCRIPT PLUS cDNA Synthesis Kit (CAT # G236, ABM) as described previously [22]. The SARS-CoV-2 viral loads were accessed using COVID-19 genesis Real-Time PCR assay on CFX96 Touch Real-Time PCR System [22] using following thermocycling conditions: 55°C for 10 min, 95°C for 2 min followed by 45 cycles of 95°C for 10s, 60°C for the 60s. The Ct values of Internal control and Target (RdRp) genes were measured on Hex and FAM channels. The Ct values were used to assess viral load in each sample [22].

A qPCR assay was used to assess the gene expression profiles of MMPs and defensins in all samples employing gene-specific primers (Table 1), while beta-actin was used as a housekeeping gene and for normalization of the gene expression data. For qPCR reaction, 2ul of cDNA was mixed with 4ul of BlasTaq 2X qPCR Master mix (Cat # G891; ABM), and 0.5ul of each reverse and forward primers. The qPCR was run using the following thermal cycling conditions: 95°C for 3 minutes, 40 cycles of 95°C for 15 seconds, and 57.8°C to 64°C (depending on the primer) for 1 minute with a melt curve at 55-95^o^C. All reactions were run in duplicate. To compare/plot the expression of each cytokine in normal versus mild, normal versus severe, and mild versus severe groups, the delta Ct method was used, while to estimate the relative expression/fold change of each MMP and defensins in the normal versus mild, normal versus severe, and mild versus severe groups, 2^(-ΔΔCt)^ methods were used [24,25].

**Table 1:** List of primers used to measure the levels of matrix metalloproteinase and defensin and β-actin.

Genes Name	Sequences 5’ – 3’	
DA 1	Fwd.: TCCCTTGCATGGGACGAAAG	Rev.: GGTTCCATAGCGACGTTCTCC
DA 3	Fwd.: TACCCACTGCTAACTCCATAC	Rev.: GAATGCCCAGAGTCTTCCC
DA 4	Fwd.: CCTTTGCATGGGATAAAAGCTCT	Rev.: ACACCACCAATGAGGCAGTTC
DA 5	Fwd.: AGACAACCAGGACCTTGCTAT	Rev.: GGAGAGGGACTCACGGGTAG
DA 6	Fwd.: CTGAGCCACTCCAAGCTGAG	Rev.: GTTGAGCCCAAAGCTCTAAGAC
MMP 1	Fwd.: AAAATTACACGCCAGATTTGCC	Rev.: GGTGTGACATTACTCCAGAGTTG
MMP 2	Fwd.: TACAGGATCATTGGCTACACACC	Rev.: GGTCACATCGCTCCAGACT
MMP 7	Fwd.: GAGTGAGCTACAGTGGGAACA	Rev.: CTATGACGCGGGAGTTTAACAT
MMP 9	Fwd.: TGTACCGCTATGGTTACACTCG	Rev.: GGCAGGGACAGTTGCTTCT
B-actin	Fwd.: CAACTTCATCCAGCTTCACC	Rev.: TCGAGGACGCCCTATCATGG

### Statistical analyses

An unpaired T-test was applied to assess statistically significant differences in viral load expression between the severe and mild groups. To compare differences in gene expression levels of matrix metalloproteinases (MMPs) and defensins among the normal, mild, and severe groups, a one-way ANOVA with Tukey’s multiple correction was applied. ANOVA was used to test the null hypothesis that there were no significant differences in expression levels between the groups. For each gene, comparisons were performed for normal vs mild, normal vs severe, and mild vs severe groups. Assumptions of normality and homogeneity of variance were tested using the Shapiro-Wilk test and Levene’s test, respectively. Results are presented as mean ± SD, and significance was set at p < 0.05. The statistical analysis was performed using R version 4.3.1 in RStudio using the R packages tidyverse, broom, and knitr. In addition, a multivariable logistic regression analysis was carried out with all the variables from the data set using ‘severity’ as the outcome variable. Regression analysis was performed using the IBM SPSS software v20. For all statistical analyses, p*<*0.05 was considered significant.

## Results

### Patient characteristics, viral load distribution in mild and severe groups, and correlation with disease severity

A total of 118 nasopharyngeal swab samples were used in this study, out of which 71 had mild and 47 had severe COVID-19 disease based on the WHO criteria [22]. Additionally, nasopharyngeal swab samples from ten normal individuals with confirmed SARS-CoV-2 negative PCR were all included in the study. Of the 47 patients with severe COVID-19 disease, 32 (68.0%) were male and 15 (31.25%) were female. The results of the descriptive statistics show that the mild group had significantly (p = 0.002) lower age (mean = 44.1 years, SD = 18.03) than the severe group (mean = 54.61 years, SD = 17.26). The mean viral loads for the mild and severe groups were 27.07 ± 5.22 and 26.37 ± 7.89, respectively; however, no association was found between viral load and disease severity (p > 0.05).

### Analysis of differential expression of defensins and MMPs in the mild and severe groups as compared to the normal group

**Fig 1:**
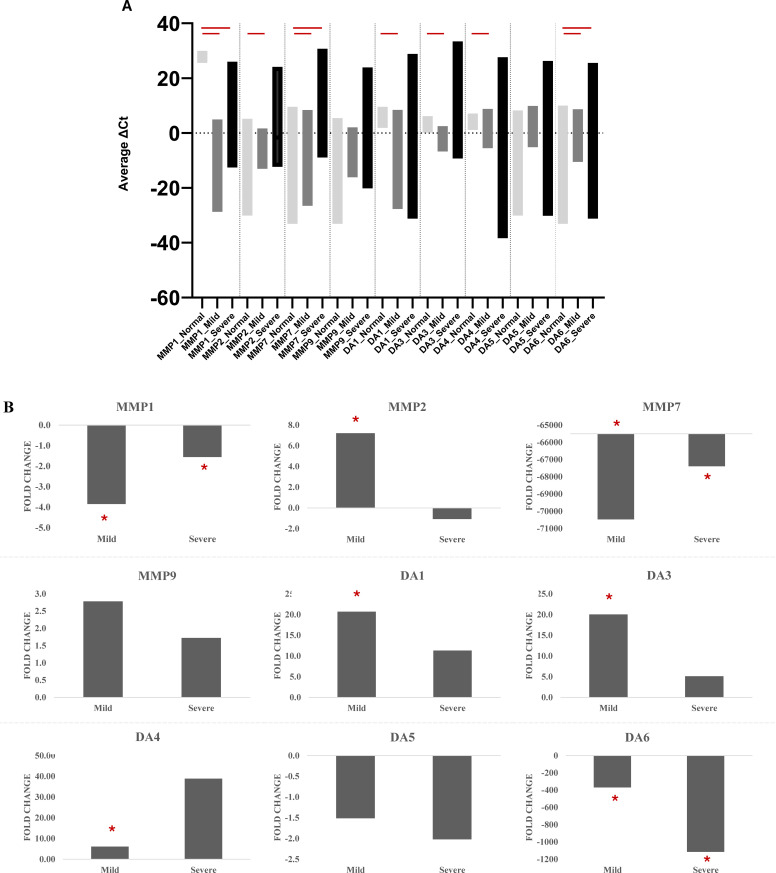
Differential gene expression profile of defensins (DAs) and matrix metalloproteinases (MMPs) in normal versus mild and severe groups. The figure shows the differential gene expression profile in mild and severe groups as A) average ΔCT values and B) fold change (2^(-ΔΔCt)^) compared to the normal group. The statistically significant (p-value <0.05) differences in the tested genes in normal vs mild vs severe groups are highlighted by a red asterisk (*) sign.

As compared to the normal group, MMP1 (Fold changes mild: -3.8, severe: -1.5; p < 0.05) and MMP7 (Fold changes mild: -70463, severe: -67387; p<0.05) exhibited decreased expression in both mild and severe groups, while MMP2 exhibited decreased expression (Fold change: 7.2; p < 0.05) in the mild group only (Fig 1A and 1B). Similarly, DA1 (Fold change: 20.7; p < 0.05), DA3 (Fold change: 20.0; p<0.05), and DA4 (Fold change: 6.08; p < 0.05) exhibited increased expression in the mild group only, while DA6 exhibited decreased expression in both mild (Fold change: -368; p<0.05) and severe (Fold change: -1114; p < 0.05) groups (Fig 1A and 1B).

### Multivariate regression analysis

Regression analysis was performed to examine the influence of all study variables with outcome ‘severity’. The results showed that the model as a whole was significant (Chi^2^(degree of freedom: 12) = 53.04, p < 0.001). Regression analysis also showed only expression of MMP1 (adjusted OR: 0.84; 95% CI: 0.73–0.98; p = 0.027), MMP2 (adjusted OR: 1.68; 95% CI: 1.27–2.22; p < 0.001) and MMP9 (adjusted OR: 0.73; 95% CI: 0.6–0.89; p = 0.002) to be significantly associated with severity (Table 2).

**Table 2:** Multivariate logistic regression model in different variables.

Variables	Odds Ratio	95% confidence interval (lower – upper)	p-values
Gender	1.03	1–1.06	0.096
Age	0.62	0.2–1.87	0.393
Viral Ct value	1.05	0.96–1.15	0.283
DA1	1.09	0.91–1.31	0.33
DA3	1.16	0.99–1.37	0.066
DA4	0.92	0.85–1	0.06
DA5	1.06	0.95–1.18	0.275
DA6	1.08	0.95–1.23	0.235
MMP1	0.84	0.73–0.98	**0.027** ^*^
MMP2	1.68	1.27–2.22	**<0.001** ^*^
MMP7	1.03	0.91–1.16	0.659
MMP9	0.73	0.6–0.89	**0.002** ^*^

## Discussion

Studies have shown that the COVID-19 disease course may vary from mild respiratory disease to severe disease with associated complications and high mortality [26]. The severity of the disease may be affected by several factors, such as the viral load, age, gender, and dysregulated expression of antiviral/proinflammatory cytokines [22,27].

A significant difference in the age of participants with mild (mean = 44.1 years, SD = 18.03) than the severe group (mean = 54.61 years, SD = 17.26) SARS-CoV-2 infection was observed, which aligns with previous research [28]. The severity of COVID-19 is influenced by age: younger infected patients had milder disease due to protective mechanisms. In contrast, older patients rely more on memory T cells for immune response, potentially leading to overreaction and tissue damage, which makes the disease more severe [27].

In our study, as compared to the normal group, the expression of DA1, DA3, and DA4 was upregulated in the mild group, whereas the expression of DA6 was downregulated in both mild and severe groups (Fig 1). Overall, defensins exhibit some degree of antiviral properties, preventing the virus from entering the cell, thereby inhibiting infection of the virus [29]. However, it is still unclear how each specific defensins affect the COVID-19 severity. Therefore, human defensins and their antiviral role are one of the major active areas of investigation [30], with studies showing defensins 4/2 to be dysregulated in COVID-19 patients [17]. Defensin 5 has also been shown to impede the entry of SARS-CoV-2 into human renal proximal tubular epithelial cells, potentially mitigating the severity of COVID-19 [31]. Studies have reported the possible inhibition of SARS-CoV-2 spike protein-mediated fusion by the defensins; for instance, HNP1 was reported to have weak inhibition of the virus attachment [32]. Furthermore, virus-mediated decreased expression of various defensin genes in COVID-19 patients may result in enhanced added bacterial infections in the upper respiratory tract; therefore, agents that can enhance the expression of human defensin genes (HBD-1-3) may have a potential therapeutic effect in these patients [33,34].

Similarly, as compared to the normal group, the expression of MMP1 and MMP7 was found to be downregulated in both mild and severe groups (Fig 1), whereas the expression of MMP2 expression was upregulated in the mild group. The role of the plasma levels of MMP2 has been reported in hypertensive COVID-19 patients [35]. Some studies state that MMP1 impacts the disease severity by damaging extracellular matrix (ECM) components that lead to tissue damage and inflammation [36,37]. On the other hand, other studies found that MMP2 is associated with severe COVID-19 due to hyperinflammation and lung tissue damage caused by the decrease in collagen levels [38]. Higher expression of MMP2 has been found in the tracheal-aspirate fluid samples of patients with severe COVID-19 patients [39]; however, we found decreased expression of MMP2 in the nasopharyngeal milieu in patients with severe COVID-19, which may suggest differences in the expression of MMPs may vary in different anatomical locations and/or cells [40,41].

Studies have shown that SARS-CoV-2 and SARS-CoV-1 use ACE2-dependent pathways involving an endosomal cathepsin protease pathway and a surface serine protease pathway [42,43]. In virus-producing cells, the proteolytic processing SARS-CoV-2 spike protein at the S1/S2 (two subunits of spike protein) boundary/cleavage site (for furin) required a higher expression of MMP9 and MMP2 proteases, which is associated with hyperinflammation and lung tissue damage in patients with COVID-19 patients [44]. Overall, in the mild group, the increased expression of DA1, 3, and 4 with antiviral effects might be associated with better clearance of the virus [45,46]. This may further be helped by decreased expression of MMP1 in mild cases, which might be related to the reduced tissue damage associated with less degradation of the extracellular matrix [9]. In severe cases, the reduced expression of DA6 may indicate dysfunctional mucosal immunity, resulting in less viral clearance in the nasal mucosal milieu [29,47].

The logistic regression analysis, where all other studied variables were examined with ‘severity’ as the outcome variable, showed that MMP1 (OR: 0.84) and MMP9 (OR: 0.73) are associated with lower odds of severity, suggesting that higher expression of these proteins may reduce disease severity. On the contrary, MMP2 (OR: 1.68) is associated with higher odds of severity, indicating that its higher expression is linked to increased disease severity. In the context of matrix metalloproteinases (MMPs), it is reported that it is an actual imbalance in the differential expression of different MMPs, leading to a dysregulated severe inflammatory status that leads to the development of severe COVID-19 disease [9,48]. Additionally, the difference between the results of fold change and the multivariate logistic regression analysis may be explained by the underlying assumptions and analytical process of each technique: fold change is a calculated parameter that shows the straightforward comparison of the gene expression levels in between groups and does not consider other possible confounding variables [49], whereas the regression analysis assesses the probability of an outcome (e.g., severity) based on multiple factors and takes into consideration their possible non-linear relationships and interactions which might be involved amongst other variables, thereby providing a more comprehensive picture [50].

We acknowledge certain limitations of our study. Firstly, the sample size was small to establish predictors of severity, however, we believe it was sufficient to show the direct correlation between the disease severity and defensins and MMP2 expression, which also agrees with the results of previous studies. Secondly, the other possible reasons for COVID-19 severity could not be established due to the non-availability of clinical information about other co-existing medical conditions that may be present in the study participants. Finally, the expression of MMPs was analyzed only in the nasopharyngeal swab samples and not in the serums, which may result in different expression profiles.

In conclusion, we found altered nasopharyngeal expression of certain defensins and MMPs in both mild and severe groups. These findings demonstrate that detection of dysregulated expression of defensins and MMPs in the nasopharyngeal milieu, which can be observed as early as three days (time of our sample collection) might be correlated with the severe form of the disease. Therefore, the early estimation of the expression of these genes may act as a useful biomarker/predictor for possible severe COVID-19 disease, which may be useful in the clinical management of patients to reduce COVID-19-associated morbidity and mortality.

## Supporting information

File S1 Data used in different analyses. (CSV)
